# Exploring the experiences of minoritised groups at life sciences conferences in the UK: new perspectives and actions to improve inclusivity in the sector

**DOI:** 10.12688/wellcomeopenres.20868.1

**Published:** 2024-03-20

**Authors:** Ireena Dutta, Michelle Bishop, Julian C. Rayner, Treasa Creavin, Saher Ahmed, Hayley Clissold, Tinu Cornish, Kathlyn Wilson, Ashlee Christoffersen, Janice Prentice, Victor Penda

**Affiliations:** 1 Wellcome Connecting Science, Hinxton, England, UK; 2 Wellcome Sanger Institute, Hinxton, England, UK; 3 Sea-Change Consultancy, London, SE10 0TS, UK

**Keywords:** Conferences, Research Meetings, Inclusion, Networking, Career Development, Knowledge Sharing, Equity

## Abstract

Attending and participating in scientific research meetings and conferences is a key mechanism for researchers to share information and knowledge, build networks, and establish relationships and collaborations to support career development. In the UK, researchers from minoritised or underrepresented groups, may have a different experience at a conference than their peers. As a high profile provider of genomics-focussed life science conferences, Wellcome Connecting Science is committed to ensuring that our events are as inclusive as possible. Here we discuss our commitments to improving access and inclusion for everyone at our scientific conferences and research meetings, and we encourage others in the sector to reflect and consider how they can also support equitable experiences.

## Plain Language Summary

Research conferences, symposia, and meetings form a key part of academic life across all subject areas from the life and physical sciences to the arts and humanities. Conferences provide a platform for sharing the latest knowledge in a specific research field, networking with colleagues, and building the personal connections that enable career development, or could lead to the creation of new projects and funding applications. Wellcome Connecting Science, part of the Wellcome Sanger Institute, is a leading provider of scientific conferences in the UK, with a focus on topics relating to genomics, health, and disease. Connecting Science has always been interested in the experiences of delegates at our meetings. We know, both from individual feedback, and longer-term analysis, that our conferences provide excellent opportunities for sharing knowledge and networking; and have led to a number of positive outcomes for event delegates. This is great news if you are ‘in the room’ and actively participating in these events; but what about those who are not in the room, and why might they be absent? We were aware that our delegate base is broadly equal in relation to gender but is not particularly diverse in relation to race and ethnicity. We worked with Sea-Change Consultancy, to explore the views and perspectives of researchers from racially minoritised backgrounds on their experiences of scientific conferences in the UK. Although there were many similarities in the barriers researchers from all backgrounds faced in accessing conferences, there were some specific issues that appeared to impact researchers from minoritised groups more than others. Based on these findings, Connecting Science is implementing an action plan to improve our conference offer, and encourages other organisations and individuals to consider how they could also improve the research conference experience for everyone.

## Disclaimer

The views expressed in this article are those of the authors. Publication in Wellcome Open Research does not imply endorsement by Wellcome.

## Introduction and context

Research conferences, symposia, and meetings form a key part of academic life across all subject areas from the life and physical sciences to the arts and humanities. Conferences provide a platform for the sharing of the latest knowledge in a specific research field; networking with colleagues and collaborators; and building the connections which enable career development, the creation of novel projects, and new funding applications. As opportunities for poster and spoken presentations are usually limited, for those selected to appear on a conference programme, this spotlight on their work can be a pivotal moment in their careers ^1^.

However, it is also clear that not everyone who attends a research conference will have the same experience, whether due to gender, disability, race and ethnicity, or the intersection of any of these, and other, characteristics. Recent studies, examining different areas of science, have indicated that women have less visibility at conferences ^2^; those from minoritised groups are less likely to be selected to present their work ^3^; and women from minoritised groups are the least likely to feel very welcome at scientific meetings, with White men as the most likely to feel this way ^4^.

In the UK, where there is a lack of diversity amongst senior leaders, we see that people of colour, and those specifically from a Black background are particularly disadvantaged, along the whole of the academic pipeline. There are degree awarding gaps at undergraduate level – students from minoritised backgrounds at UK universities are less likely than their White counterparts to graduate with a higher classification from undergraduate programmes ^5^; there are proportionally fewer Black people at postgraduate and professorial levels ^6^; and Black researchers have lower success rates with funding councils and granting agencies ^7^. These known structural barriers can be further compounded when specific groups miss out on the full research conference experience.

## Aims

Wellcome Connecting Science is a high-profile provider of life science conferences in the UK. As part of the same Wellcome-funded organisation as the Wellcome Sanger Institute, its goal is to enable everyone to explore the impact of genomics on research, health, and society. As attendance at scientific conferences can be very influential in relation to career development, Connecting Science sought to understand more about how its research meetings could positively contribute to addressing the lack of academic equity in the UK, and influence change in this sector. The Connecting Science conference attendee base is broadly gender balanced, with the majority of attendance from institutions in the UK and Europe. But there is likely to be a lack of representation of researchers from minoritised groups based on race and ethnicity. So, this cohort of researchers is not only potentially missing out on scientific content, but also the career enhancing impact conferences can have through the contacts, ideas and collaborations that are generated there.

The conference delegate feedback Connecting Science receives for its events is overwhelmingly positive. It indicates a high-level of satisfaction with the overall experience; with events meeting expectations around knowledge sharing, collaboration, and career development. But the programme acknowledges that this only reflects the perspectives of those who are ‘in the room’ and attending meetings. Connecting Science wanted to improve its understanding of who wasn’t in the room, and why this might be. This project was therefore centred on the conference experiences of researchers from minoritised groups in the UK, with a particular focus on race and ethnicity. The project aimed to provide insights into the experiences of researchers from minoritised backgrounds who had attended any life science conference in the UK (including those delivered by Connecting Science); and the experiences of researchers from minoritised backgrounds who had not attended a Connecting Science conference, and why this was; and were there any barriers preventing or discouraging attendance that Connecting Science could seek to address?

The overall goal of this work was to use the identified insights to enhance the Connecting Science conference offer, and to influence change in the wider UK conferencing sector, in order to make the research conference experience a more equitable one for everyone.

## Summary of insights

In order to obtain an independent perspective on researcher experiences, Connecting Science commissioned Sea-Change Consultancy, a group focussed on equality, diversity and inclusion in an organisational change context, to explore the following questions between February - November 2022.

- What are researchers’ experiences at conferences? Is there a difference between the experiences of minoritised researchers and majority groups?
- What are the barriers to attending a research conference? What elements support attendance, especially attendance of researchers from minoritised groups?
- What are the positive impacts of attending a conference? Are the impacts the same for minoritised researchers and majority groups?
- What practical steps have conference organisers taken to improve experiences for minoritised researchers?

Researchers from ethnically minoritised and non-minoritised groups, with a range of seniority and institutional affiliations in the UK, participated in this project, contributing their views and experiences.

There were broadly no major differences between ethnically minoritised and White researchers in the discussion and responses to these questions, suggesting that there are potentially more differences across role and career stage, rather than ethnic group in relation to conference access and experience. For example, institutional barriers relating to funding, lack of time, or varying levels of support from supervisors were consistent themes. However, there were some notable areas of difference between groups.

- Access to financial support and caring responsibilities as barriers to conference attendance, appeared to disproportionately impact researchers from ethnically minoritised backgrounds, in comparison to their White peers.
- A lack of knowledge and awareness of research meetings was also a possible barrier, with proportionately more White researchers finding out about conferences from colleagues and through word of mouth. This potentially supports a general pattern of exclusion of those who are not part of these formal and informal networks.
- Some researchers described uncomfortable experiences based on their ethnicity and/or gender; with some feeling that the intersection of these rather than a specific attribute may have been a factor; others described the sense of isolation felt when they were the only woman, or Black person, in the room.
- Ethnically minoritised researchers were less likely to agree that they felt welcomed and included at life science conferences compared to White researchers.

The full report detailing the project findings can be downloaded from the Wellcome Connecting Science website: https://www.wellcomeconnectingscience.org/news/new-insights-into-the-experiences-of-different-groups-at-research-conferences-in-the-uk/

## Next steps and actions

It is clear from the findings of this project, the existing literature, and anecdotal feedback from peers, colleagues, and our programme participants, that attendance and participation in a research conference can be a highly positive and beneficial experience. Wellcome Connecting Science develops and delivers conferences as part of the research and healthcare professional community in the UK, and impact on knowledge sharing and career development is a key motivator in the programme continuing to support these events.

But it is also evident that not everyone is always able to access conference events, or experience these events in an equitable manner; and that the experiences of different groups of researchers can vary significantly. Where there are barriers to access, they often disproportionately impact researchers who are from ethnically minoritised backgrounds in the UK.

However, many common themes around access also emerged, based on the shared experience of being an early career researcher, with limited resources and time. By recognising and acknowledging these different types of experiences, Connecting Science aspires to enhance its own conference offer; and improvements to inclusion and participation will benefit not just minoritised groups, but everyone in the research community.

Connecting Science has committed to a number of actions, based on the findings of this project:

- Creating a new and intersectional inclusion policy to diversify conference participation - by clearly articulating its strategic goals and commitments, this policy will set the ethos of the Connecting Science conference offer.
- Enhancing the Connecting Science Conference Code of Conduct - clear expectations around acceptable and unacceptable behaviour, and how to access help and support, will contribute to conference attendees feeling safe and comfortable in this environment, improving their ability to learn and network.
- Improving how Connecting Science promotes its financial support options to specific target groups - there are several support opportunities available, but ensuring that these are pro-actively and consistently communicated, will become a priority.
- Ensuring Connecting Science marketing and communication uses inclusive approaches - both imagery and language will be regularly reviewed to ensure it is inclusive and comprehensible.

Connecting Science acknowledges that the impact of these actions may be challenging to identify, but through its event-specific evaluation and feedback, it will actively monitor any potential change in the participant experience, or the composition of the conference delegate base. Connecting Science is not proposing that its actions provide a solution that will fully address current differences in experience, but is sharing its plans to prompt further thought and reflection across the sector.

This project aims to act as a call to action to everyone involved in research conferences, whether as participants or as organisations. Conferences are valuable because they reflect an academic collective with a shared endeavour; and being part of that community generates new thinking, ideas, and opportunities. Connecting Science believes that all researchers should be able to enjoy these benefits in as equitable a way as possible, and that we all have a role to play in this.

As a programme that is strongly linked to Wellcome Sanger Institute science, and focussed on delivering excellent learning and training outcomes for research and healthcare professionals, Connecting Science has a significant degree of influence as well as the ability to shape and alter its conference offer. But it is also committed to sharing what it has learnt from this project, to contribute to the development of the life science conference sector as a whole. Connecting Science encourages other conference providers to consider how their events are accessed and experienced, and how this could be enhanced to support equitable participation.

Connecting Science also recognises the influential role that individual research professionals can have in this sphere. As committee members on conference organising committees, research and healthcare professionals are generally responsible for the provision of intellectual input and ideas, and play a pivotal role in creating engaging and stimulating meetings. As conference attendees, research and healthcare professionals are also able to influence how meetings are delivered - making decisions on where to allocate their registration fee budget. Connecting Science encourages all research professionals to consider how conference providers (including Connecting Science itself) ensure that their events are equitable for all participants, and where appropriate, to challenge the conference sector to do better.

## Data Availability

No data are associated with this article.
