# Exploring the experiences of minoritised groups at life sciences conferences in the UK: new perspectives and actions to improve inclusivity in the sector

**DOI:** 10.12688/wellcomeopenres.20868.2

**Published:** 2024-11-20

**Authors:** Ireena Dutta, Michelle Bishop, Julian C. Rayner, Treasa Creavin, Saher Ahmed, Hayley Clissold, Tinu Cornish, Kathlyn Wilson, Ashlee Christoffersen, Janice Prentice, Victor Penda

**Affiliations:** 1 Wellcome Connecting Science, Hinxton, England, UK; 2 Wellcome Sanger Institute, Hinxton, England, UK; 3 Sea-Change Consultancy, London, SE10 0TS, UK

**Keywords:** Conferences, Research Meetings, Inclusion, Networking, Career Development, Knowledge Sharing, Equity

## Abstract

Attending and participating in scientific research meetings and conferences is a key mechanism for researchers to share information and knowledge, build networks, and establish relationships and collaborations to support career development. In the UK, researchers from minoritised or underrepresented groups may have a different experience at a conference than their peers. As a high profile provider of genomics-focussed life science conferences, Wellcome Connecting Science is committed to ensuring that our events are as inclusive as possible. Here we summarise the findings of a project to explore the experiences of minoritised groups, with a focus on race and ethnicity, and discuss how we are seeking to improve access and inclusion at our conferences. , We

## Plain Language Summary

Research conferences, symposia, and meetings form a key part of academic life across all subject areas from the life and physical sciences to the arts and humanities. Conferences provide a platform for sharing the latest knowledge in a specific research field, networking with colleagues, and building the personal connections that enable career development, or could lead to the creation of new projects and funding applications. Wellcome Connecting Science, part of the Wellcome Sanger Institute, is a leading provider of scientific conferences in the UK, with a focus on topics relating to genomics, health, and disease. Connecting Science has always been interested in the experiences of delegates at our meetings. We know, both from individual feedback, and longer-term analysis, that our conferences provide excellent opportunities for sharing knowledge and networking; and have led to a number of positive outcomes for event delegates. This is great news if you are ‘in the room’ and actively participating in these events; but what about those who are not in the room, and why might they be absent? We were aware that our delegate base is broadly equal in relation to gender but is not particularly diverse in relation to race and ethnicity. We worked with Sea-Change Consultancy, to explore the views and perspectives of researchers from racially minoritised backgrounds on their experiences of scientific conferences in the UK. Although there were many similarities in the barriers researchers from all backgrounds faced in accessing conferences, there were some specific issues that appeared to impact researchers from minoritised groups more than others. Based on these findings, Connecting Science is implementing an action plan to improve our conference offer, and encourages other organisations and individuals to consider how they could also improve the research conference experience for everyone.

## Disclaimer

The views expressed in this article are those of the authors. Publication in Wellcome Open Research does not imply endorsement by Wellcome.

A note on language: Where we use the terms Black and White in this article, we intend this to mean ‘racialised as Black’ or ‘racialised as White’ to counter the perception of White as a neutral baseline. People of colour form the global majority, and in many contexts this descriptor is relevant and helpful. However in the context of the UK, and for the purposes of this article, we use the term ethnically minoritised researchers to describe researchers who self-identify their ethnicity as other than the majority group in the UK (‘White English/Welsh/Scottish/Northern Irish or British’). Where relevant we describe specific minority communities, and also recognise that minoritised communities in the UK are not a homogenous group.

## Introduction and context

Research conferences, symposia, and meetings form a key part of academic life across all subject areas from the life and physical sciences to the arts and humanities. Conferences provide a platform for the sharing of the latest knowledge in a specific research field; networking with colleagues and collaborators; and building the connections which enable career development, the creation of novel projects, and new funding applications. As opportunities for poster and spoken presentations are usually limited, for those selected to appear on a conference programme, this spotlight on their work can be a pivotal moment in their careers ^1^.

However, it is also clear that not everyone who attends a research conference will have the same experience, whether due to gender, disability, race and ethnicity, or the intersection of any of these, and other, characteristics ^2^. Recent studies, examining different areas of science, have indicated that women have less visibility at conferences ^3^; those from minoritised groups are less likely to be selected to present their work ^4^; and women from minoritised groups are the least likely to feel very welcome at scientific meetings, with White men as the most likely to feel this way ^5^.

In the UK, where there is a lack of diversity amongst senior leaders in research and academia, we see that people of colour (the global majority), and those specifically from a Black background are particularly disadvantaged, along the whole of the academic pipeline ^6^. There are degree awarding gaps at undergraduate level – students from minoritised backgrounds at UK universities are less likely than their White counterparts to graduate with a higher classification from undergraduate programmes ^7^; there are proportionally fewer Black people at postgraduate and professorial levels ^8^; and Black researchers have lower success rates with funding councils and granting agencies ^9^. In addition, specific British South Asian communities of Pakistani and Bangladeshi heritage, encounter similar disadvantage. A fact recently recognised by the funder, Wellcome, through a new funding award scheme targeted at these groups ^10^.

These known structural barriers can be further compounded when specific groups miss out on the full research conference experience.

## Aims

Wellcome Connecting Science is a high-profile provider of life science conferences in the UK. As part of the Wellcome-funded organisation, the Wellcome Sanger Institute, our goal is to enable everyone to explore the impact of genomics on research, health, and society. Connecting Science sought to understand more about how its research meetings could positively contribute to addressing the lack of academic equity in the UK, and influence change in this sector. We undertook this work, not as a research project, but as an insights-based activity that could be directly translated into operational actions.

The Connecting Science conference attendee base is broadly gender balanced, with the majority of attendance from institutions in the UK and Europe. But there is likely to be a lack of representation of researchers from minoritised groups based on race and ethnicity. So, this cohort of researchers is not only potentially missing out on scientific content, but also the career enhancing impact conferences can have through the contacts, ideas and collaborations that are generated there.

The conference delegate feedback Connecting Science receives for its events is overwhelmingly positive. It indicates a high-level of satisfaction with the overall experience; with events meeting expectations around knowledge sharing, collaboration, and career development. But we acknowledge that this only reflects the perspectives of those who are ‘in the room’ and attending meetings. This activity was therefore centred on the conference experiences of researchers from minoritised groups in the UK, with a particular focus on race and ethnicity. The activity had two main objectives:

i) For researchers in the UK minoritised in relation to race and ethnicity, who had **not** attended a life science conference – to provide information and insights into the factors and barriers that prevented their attendance.
ii) For researchers in the UK minoritised in relation to race and ethnicity, who **had** attended a life science conference – to provide information and insights into their experiences at these events.

For both of these objectives, we were interested in experiences and insights that were based on our own events, and the broader scientific conferencing sector in the UK.

Our goal was to use these insights to reduce barriers which may prevent or discourage attendance; enhance factors which might improve the conference experience; and to influence change in the wider UK conferencing sector, adding to prior thinking in this area ^11^. 

## Summary of insights

In order to obtain an independent perspective on researcher experiences, Connecting Science commissioned Sea-Change Consultancy, a group focussed on equality, diversity and inclusion in an organisational change context, to explore the following questions between February - November 2022.

- What are researchers’ experiences at conferences? Is there a difference between the experiences of minoritised researchers and majority groups?
- What are the barriers to attending a research conference? What elements support attendance, especially attendance of researchers from minoritised groups?
- What are the positive impacts of attending a conference? Are the impacts the same for minoritised researchers and majority groups?
- What practical steps have conference organisers taken to improve experiences for minoritised researchers?

Researchers from ethnically minoritised and non-minoritised groups, with a range of seniority and institutional affiliations in the UK, participated in this project, contributing their views and experiences. These were gathered via an online survey and a series of interviews. The survey generated 55 responses, and of these 16 participants were interviewed in further detail. We acknowledge that these ‘traditional’ approaches can vary in effectiveness, perhaps reflected in the small survey sample, and risk response bias. However, we believe that the perspectives drawn from this group provided pertinent insights.

Full details of the approach are included in the final report which can be downloaded from the Wellcome Connecting Science website: https://www.wellcomeconnectingscience.org/news/new-insights-into-the-experiences-of-different-groups-at-research-conferences-in-the-uk/

Broadly, there were many similarities in discussion within research role or career stage groups. For example, institutional barriers relating to funding, lack of time, or varying levels of support from supervisors were consistent themes. But what also emerged was the intersection of identities, amplifying or adding to challenges experienced by ethnically minoritised researchers, for example when they were also women, and/or early career researchers. And there were some notable areas of difference between groups.

- Access to financial support and caring responsibilities as barriers to conference attendance, appeared to disproportionately impact researchers from ethnically minoritised backgrounds, in comparison to their White peers.
- A lack of knowledge and awareness of research meetings was also a possible barrier, with proportionately more White researchers finding out about conferences from colleagues and through word of mouth. This potentially supports a general pattern of exclusion of those who are not part of these formal and informal networks.
- Some researchers described uncomfortable experiences based on their ethnicity and/or gender; with some feeling that the intersection of these rather than a specific attribute may have been a factor; others described the sense of isolation felt when they were the only woman, or Black person, in the room.
- Ethnically minoritised researchers were less likely to agree that they felt welcomed and included at life science conferences compared to White researchers.

The full report detailing the findings can be downloaded from the Wellcome Connecting Science website: https://www.wellcomeconnectingscience.org/news/new-insights-into-the-experiences-of-different-groups-at-research-conferences-in-the-uk/

## Next steps, recommendations and actions

It is clear from the findings of this work, the existing literature, and anecdotal feedback from peers, colleagues, and our programme participants, that attendance and participation in a research conference can be a highly positive and beneficial experience. It is also evident that not everyone is always able to access conference events, or experience these events in an equitable manner. The experiences of different groups of researchers can vary significantly, andbarriers often disproportionately impact researchers who are from ethnically minoritised backgrounds in the UK. Many common themes around access also emerged, based on the shared experience of being an early career researcher or a woman. By recognising and acknowledging these different types of experiences, Connecting Science aspires to enhance its own conference offer; making improvements to inclusion and participation that will benefit not just groups minoritised in relation to race and ethnicity in the UK, but everyone in the research community.

This work delivered 24 recommendations, which are available in the full report. We highlight below the recommendations that we have prioritised as part of a Diversity and Inclusion Action Plan. Our focus, presented in the table below, has been on addressing areas of difference between those minoritised in relation to race and ethnicity and their White peers, with relevant actions to reduce these differences.

**Table:** 

Area of difference identified between majority and minority researchers	Recommendation	Connecting Science action
Financial support and caring responsibilities	Provide and promote targeted bursaries for researchers from ethnically minoritised groups to attend. Promote the support available for researchers with caring responsibilities.	We provide several financial and caring responsibility support options available, but ensuring that these are pro-actively and consistently communicated to target groups, will become a priority.
Knowledge and awareness of research meetings	Include content that is specifically designed to attract and address the priorities, concerns, and needs of ethnically minoritised individuals. Increase the proportion of images of ethnically minoritised individuals, attending to the intersectionality between race and gender when choosing images.	Ensuring Connecting Science marketing and communication uses inclusive approaches - both imagery and language - will be regularly reviewed to ensure it is inclusive and comprehensible.
Uncomfortable experiences based on ethnicity and/or gender; and feeling welcome and included	Produce guidelines for the organisation of diverse and inclusive conferences, making compliance with these guidelines a prerequisite for being part of the WCS conference programme.	Creating a new and intersectional inclusion policy to support conference participation - by clearly articulating our strategic goals and commitments, this policy will set the ethos of the Connecting Science conference offer, and will be visible on our website and other materials, for all conference participants, speakers, and committee members. Enhancing the Connecting Science Conference Code of Conduct – setting clear expectations around acceptable and unacceptable behaviour, and how to access help and support, will contribute to conference attendees feeling safe and comfortable in this environment. The Code of Conduct will be clearly visible on our website and other materials, and our team will be trained and supported in its implementation.

In addition, we will seek to reduce the impact of ‘gatekeeping’ on decisions around abstract selection and conference poster presentations, by introducing a requirement for decision-makers (for example scientific committee members) to participate in unconscious bias training. A new registration platform will underpin many of these actions, enhancing our capability to capture and analyse diversity and inclusion-related data, in a manner that has not previously been possible.

Connecting Science acknowledges that the impact of these actions may be challenging to identify, but through event-specific evaluation and feedback, we will actively monitor change in the participant experience, and the composition of the conference delegate base. Connecting Science is not proposing that its actions provide a solution that will fully address current differences in experience, but is sharing its plans to prompt further thought and reflection across the sector.

As a programme that is strongly linked to Wellcome Sanger Institute science, we have a significant degree of influence as well as the ability to shape and alter our conference offer. This article aims to act as a call to action to everyone involved in research conferences, whether as organisations or participants. Connecting Science also recognises the influential role that individual research professionals can have in this sphere. As committee members on conference organising committees, research and healthcare professionals are generally responsible for the provision of intellectual input and ideas, and play a pivotal role in creating engaging and stimulating meetings. As conference attendees, research and healthcare professionals are able to influence how meetings are delivered - making decisions on where to allocate their registration fee budget. We encourage all research professionals to consider how conference providers (including Connecting Science itself) ensure that their events are equitable for all participants, and where appropriate, to challenge the conference sector to do better.

## Data Availability

No data are associated with this article.
