# Supplementary material for: Exploring the experiences of minoritised groups at life sciences conferences in the UK: new perspectives and actions to improve inclusivity in the sector
Source: Wellcome Open Res. 2025 Jan 15;9:166. Originally published 2024 Mar 20. [Version 3] doi: 10.12688/wellcomeopenres.20868.3 (PMC11748436; doi:10.12688/wellcomeopenres.20868.3)
Supplement: Peer review history

# Reviewer report: Reviewer response for version 3

**DOI:** 10.21956/wellcomeopenres.26060.r117764

**Published:** 2025-03-06

**Authors:** Joe Yates

**Affiliations:** 1 London School of Hygiene & Tropical Medicine (LSHTM), London, UK

This Open Letter covers an important and still underserved topic of inequities in scientific conference participation. It presents a very high level summary of a separate report that was conducted by a consultancy organisation called Sea Change. The role of the reviewer as I have been instructed and understand it is to simply review the letter.

 Taking it in its intended format, the letter is informative, useful and practical. It draws attention to the multiple marginalisations (which in many case act upon one individual) that form barriers to meaningful participation in scientific fora. I appreciated the way that, in plain language, it described this concept of intersectionality.  

 The task to review this was slightly challenging, because it feels like a piece of research, yet is presented as an “insights-based activity”. As such, some of the questions you might usually pose as a reviewer (i.e. what is the sample size, is there risk of non-response bias etc.) feel overly robust. However, it does seem important to note that its hard to know whether the sample size is relatively small or not because – as far as I can see from the report (which I did scan) – this number is not contextualized as a % of the total participants. This makes it a bit difficult to consider the strength and representativeness of the sample.

 Despite this, I think the piece is definitely worthy of indexing. Small qualitative samples can tell us a lot about common barriers and constraints that persist across society. Although it was beyond the remit of my review, I did read some of the quotes in the report and found them to be very insightful and thought provoking.

 Scientific conferences do not sit in a vacuum. They reflect the way wider society is organised. Although it is brief, this letter does a good job of conveying this. I liked the practical recommendations for conference organisers, who as the authors rightfully state, hold a lot of power. It would have been good to see a few more recommendations and for the piece to engage in more detail about the specifics of implementing these and why they could make a material difference. But I appreciate that these are in the full report.

 I think there could be scope to discuss different conference formats also. Apparently (again, according to the report, which was technically out of my remit!) the Connecting Science event is available both online and in person. This is quite an important point to cover, as these formats and the many different ways they can be curated bring a wide range of benefits and challenges for different population subgroups.  Even just a single sentence along with 1-2 citations to peer reviewed evidence to acknowledge this could be good, since I would hope that conference organisers will read the Open Letter.

 Overall, I’m glad to have been invited to read this piece and appreciate the work done by the authors to raise attention to this critical issue.

**Some small specific thoughts:**

- Sample of participants. It would be good to know the total number of conference participants, which would help to place the 55 respondents into context. A simple number and % would do.

 

- It might be worth adding “and whom were motivated to complete the feedback survey” to the end of this passage: *“But we acknowledge that this only reflects the perspectives of those who are ‘in the room’ and attending meetings”*

 

- I really liked the third and second paragraphs towards the end which discuss abstract selection committees and reflects on some ways that Connecting Science can address biases. Again, there is a lot more to say on this topic i.e. the way Call for Abstracts are designed and disseminated, the diversity of members on a Committee, the guidance they get, the design of abstract review forms etc. This section suffers a little due to the slightly confusing format of an Open Letter, which leaves the reader wanting more nuance i.e. through engaging with wider literature etc., while also recognising that it’s not within scope. Maybe this is more of a suggestion for Wellcome Open Research than the authors.

Does the article adequately reference differing views and opinions?

Yes

Are all factual statements correct, and are statements and arguments made adequately supported by citations?

Yes

Is the Open Letter written in accessible language?

Yes

Where applicable, are recommendations and next steps explained clearly for others to follow?

Yes

Is the rationale for the Open Letter provided in sufficient detail?

Yes

Reviewer Expertise:

Interdisciplinary capability sharing, sustainable and equitable conferences, political economy of plastics and food systems,

I confirm that I have read this submission and believe that I have an appropriate level of expertise to confirm that it is of an acceptable scientific standard.

# Reviewer report: Reviewer response for version 3

**DOI:** 10.21956/wellcomeopenres.26060.r117762

**Published:** 2025-02-12

**Authors:** Martin Thomas Falk

**Affiliations:** 1 University of South-Eastern Norway, Gullbringvegen, Norway

I understand this is not a research article but an open letter with insights that may be useful to others in this field. Please see my comments.

**Main comments**

 1. The literature review must be updated. There are very few references from academic journals 

 2. What about online conferences? Online conferences allow minorities easy access. This should be discussed. 

 3. More information on the data collection is needed (consent of the respondents, characteristics of the respondents).’The survey is small and not representative. 

 4. Compare the empirical findings of your survey with the previous literature 

 5. Avoid self-promotion of your research. Why do you only focus on the initiatives of “Wellcome Connecting Science”? Many scientific associations organising conferences have changed their statutes towards gender representation and inclusion of minorities.

**Minor comments**

 A. “ Conferences provide a platform for the sharing of the latest knowledge in a specific research field; networking with colleagues and collaborators; and building the connections which enable career development, the creation of novel projects, and new funding applications. “

 A citation is required here 

 B. Perhaps you use the term WEIRD (Western, Educated, Industrialized, Rich, and Democratic)

 C. Consider a link to the sustainable development goals .

Does the article adequately reference differing views and opinions?

No

Are all factual statements correct, and are statements and arguments made adequately supported by citations?

No

Is the Open Letter written in accessible language?

Yes

Where applicable, are recommendations and next steps explained clearly for others to follow?

Partly

Is the rationale for the Open Letter provided in sufficient detail?

No

Reviewer Expertise:

Quantitative research methods, Social Sciences

I confirm that I have read this submission and believe that I have an appropriate level of expertise to state that I do not consider it to be of an acceptable scientific standard, for reasons outlined above.

# Reviewer report: Reviewer response for version 3

**DOI:** 10.21956/wellcomeopenres.26060.r117290

**Published:** 2025-02-10

**Authors:** Susanne Beck, Thomas Palfinger

**Affiliations:** 1 Warwick Business School, Coventry, UK; 2 LBG Open Innovation in Science (OIS) Center, Vienna, Austria

The authors have done an excellent job in revising their open letter. It has significantly improved, and we sincerely appreciate the effort and thought that went into the revisions. The purpose and objectives of the letter are now much clearer, making it a stronger and more compelling piece.

 Before changing the status, we have a few suggestions that could further enhance the impact of your work:

- You mention (in version 2) that gender is not a primary focus, yet the results suggest it plays a role. Have you considered including women in the category of “minoritized” individuals as well? This might strengthen your argument and ensure broader inclusivity. You may have already made this change in Version 3. 
- The recommendations could benefit from additional specificity. For instance, you mention support for researchers with caring responsibilities—could you provide concrete examples of how this support is structured? Similarly, what processes are in place to determine what content aligns with the “priorities, concerns, and needs of ethnically minoritized individuals”? Additionally, how was the Code of Conduct developed to actively foster a welcoming environment for these groups?
- More broadly, it would be helpful to understand the mechanisms through which your recommendations address the identified challenges. For example, how does increasing the representation of minoritized individuals in images contribute to improving “Knowledge and awareness of research meetings”? Likewise, how does the presence of a Code of Conduct make a tangible difference for minoritized groups?
- Regarding data collection, you propose gathering more information at submission to capture diversity and training decision-makers in unconscious bias, rather than anonymizing all identifiable data. Could you expand on the rationale behind this approach and provide further justification?

Overall, the letter serves as a strong call to action, but some areas could benefit from additional clarity regarding implementation. We see two possible ways forward:

  

1. Minor revision: Adjust the table so that the problem (column 1) is more explicitly stated, the recommended measure (column 2) is made more concrete with a clear rationale for its effectiveness, and the concrete actions (column 3) are elaborated in more detail.
2. Major revision: You could reduce the space dedicated to describing the problem—since this is an important issue most researchers would agree on—and focus more on actionable solutions. Specifically, which problems need to be addressed, what measures do you propose, and why do you believe they will be effective?

We appreciate the work you’ve put into this revision and look forward to seeing how you further refine this important publication!

Does the article adequately reference differing views and opinions?

Partly

Are all factual statements correct, and are statements and arguments made adequately supported by citations?

Partly

Is the Open Letter written in accessible language?

Yes

Where applicable, are recommendations and next steps explained clearly for others to follow?

Partly

Is the rationale for the Open Letter provided in sufficient detail?

Yes

Reviewer Expertise:

Susanne's research focuses on the organization of science, managing open and collaborative scientific knowledge production, Open Innovation in Science, and the role of AI in Science; Thomas has experiences in designing co-creation processes and foster public involvement in science. In addition, We have substantial experience in organizing scientific conferences.

We confirm that we have read this submission and believe that we have an appropriate level of expertise to confirm that it is of an acceptable scientific standard, however we have significant reservations, as outlined above.

# Reviewer report: Reviewer response for version 3

**DOI:** 10.21956/wellcomeopenres.26060.r117291

**Published:** 2025-01-17

**Authors:** Sara Lil Middleton

**Affiliations:** 1 University of Oxford, Oxford, England, UK

I have now read version 3 of this article, and I am really pleased with the changes the authorship team have made with regards to research ethics in the Aims section. The last two paragraphs of the Aims section now clearly communicate the steps taken to ensure ethical standards were met.

 I am satisfised that the points raised in my first and second reviewer report have been addressed and can now confidently approve the paper!

Does the article adequately reference differing views and opinions?

Partly

Are all factual statements correct, and are statements and arguments made adequately supported by citations?

Yes

Is the Open Letter written in accessible language?

Yes

Where applicable, are recommendations and next steps explained clearly for others to follow?

Partly

Is the rationale for the Open Letter provided in sufficient detail?

Yes

Reviewer Expertise:

Justice, equity, diversity and inclusion in STEM and academia; intersectionality, bias, discrimination in academia with focus on race, gender, disability, and neurodiversity.

I confirm that I have read this submission and believe that I have an appropriate level of expertise to confirm that it is of an acceptable scientific standard.

# Reviewer report: Reviewer response for version 2

**DOI:** 10.21956/wellcomeopenres.25828.r112473

**Published:** 2024-12-03

**Authors:** Sara Lil Middleton

**Affiliations:** 1 University of Oxford, Oxford, England, UK

The revised version is much improved in terms of clarity, particularly in the use of language and the addition of the table in “Next steps, recommendations and actions” section. Well done to the authors!

 The only point that was not fully clarified in both the author response or revised version is a statement about research ethics. A clarifying statement is needed as to how the authors followed ethical research practices given personal information from participants was gathered from surveys and interviews (even if data were anonymized for report/summary article).

 Very often studies involving human participants have a short statement like this:

 Ethical approval was obtained by [ ….. ] ethics panel for this study (approval [ …..]). All participants provided written informed consent. Personal information obtained from surveys and interviews were anonymized and stored securely.

 The article needs to have explicit mention of how the authors followed ethical research practices or a statement that ethical approval was not required for the study. This can be written in the “Summary of insights” and a separate “Consent” statement at the end of the article. For guidance, please see section 4 of Ethical Policies, Research involving humans: https://wellcomeopenresearch.org/about/policies/#ethpol

 I did also notice two typos that need fixing:

 Abstract:  At the end of the abstract “, We” appears (remove or complete the sentence)

 Next steps, recommendations and actions: “and barriers” needs a space.

 The article is much stronger now and I commend the authors for their work on taking on board reviewer feedback. I would like to see the point about research ethics addressed before I change the approval status of the article. Looking forward to seeing the revised version in due course.

Does the article adequately reference differing views and opinions?

Partly

Are all factual statements correct, and are statements and arguments made adequately supported by citations?

Yes

Is the Open Letter written in accessible language?

Yes

Where applicable, are recommendations and next steps explained clearly for others to follow?

Partly

Is the rationale for the Open Letter provided in sufficient detail?

Yes

Reviewer Expertise:

Justice, equity, diversity and inclusion in STEM and academia; intersectionality, bias, discrimination in academia with focus on race, gender, disability, and neurodiversity.

I confirm that I have read this submission and believe that I have an appropriate level of expertise to confirm that it is of an acceptable scientific standard, however I have significant reservations, as outlined above.

# Reviewer report: Reviewer response for version 1

**DOI:** 10.21956/wellcomeopenres.23090.r87540

**Published:** 2024-08-06

**Authors:** Susanne Beck, Thomas Palfinger

**Affiliations:** 1 Warwick Business School, Coventry, UK; 2 LBG Open Innovation in Science (OIS) Center, Vienna, Austria

The timely and relevant open letter explores barriers to access and participation in academic conferences for minoritized groups, specifically focusing on racial and ethnic minorities. It aims at examining existing challenges and proposes actionable strategies to enhance inclusivity in scientific gatherings. The open letter aims to provide guidelines and an action plan to improve conference experiences and opportunities for underrepresented researchers.

 The well-written article addresses relevant topics but would benefit from further revisions to enhance clarity and impact. In particular, based on our reading, there are two main aspects that would deserve more attention and which are detailed in the following comments. First, the objectives presented in the frontend are not consistently reflected in the remaining letter. Second, the insights presented in the letter do not align well with the actions outlined in the end. Given the importance of the topic, we hope our comments detailed below  encourage discussions among the authors to achieve the best possible outcome for the community.

 We understand that the format necessitates a concise presentation of messages and knowledge. However, in some sections, such as "Summary of Insights" and "Next Steps and Actions" (refer to Lyn Horn's review), significant content reduction harms the message and can sometimes lead to apparent contradictions. Simultaneously, other aspects, like the general importance of conferences for scientists, are repetitively covered, occupying a disproportionate amount of space. The letter could focus more clearly on practical applications to provide the potential target audience (e.g., conference organizers) with clear messages and actionable recommendations.

 In many points, we agree with the other reviewers. The methodology could be presented in more detail and more clearly. We align with Lyn Horn’s review regarding the difficulty in extracting the sample size (N) from the linked report, and the letter’s informative value would benefit from describing the methods. Additionally, we encourage the authors to reflect on the difficulty of reaching the target group (minoritized groups) using traditional methods (surveys or interviews). It's commendable that this was attempted, and sharing this experience with readers would be valuable. For readers, it might also be helpful to include references to existing sources with good/best practices (see suggestions from Reviewer 1 and 2, Sara Middleton & Lyn Horn).

 Besides the methodology, the letter would benefit from a clearer formulation of its objectives and more consistent pursuit of these goals. The two abstracts point in different directions, which is reflected in various sections of the article (the short abstract suggests disadvantages for minoritized groups during conference participation while the abstract in plain language suggest barriers to conference participation). It is also unclear who exactly falls under the term "minoritized group" (ethnic, disabled, racial, etc.). The core objective probably is making conferences more inclusive. But issues like the lack of diversity among senior leaders or problems with inclusion in study programs are also raised, which are societal issues rather than conference-specific ones. These topics are undoubtedly relevant and should be addressed, but perhaps go beyond the scope of  this letter. Hence, to emphasize the core message of the letter, it would be helpful to 1) clarify whether the main goal is increase access to conferences (entry barriers) or the accessibility of conference benefits (key mechanisms); and 2) to clearly define what is meant with minoitized groups. Please also note, that there is a large variety across minoritized groups and they should not be falsely considered as one homogeneous group.

 The briefly outlined actions at the end of the letter do not sufficiently reflect earlier discussions of the identified (or hinted) problems of minoritized groups. For instance, an inclusion policy or a code of conduct targeting conference organizers was not mentioned as a lever before. Similarly, financial support would benefit everyone equally instead of specifically reducing disadvantages for minoritized groups. Strengthening the link between insights and action plan would provide a more consistent picture.

 Overall, the letter should be reviewed for its internal logic (i.e., the line of arguments) to reduce what appears to be contradictions. For example, in the second paragraph of the "Next Steps and Actions" section, the letter states: “Where there are barriers to access, they often disproportionately impact researchers who are from ethnically minoritised backgrounds in the UK”, which contradicts the earlier statement “There were broadly no major differences between ethnically minoritised and White researchers in the discussion and responses to these questions, suggesting that there are potentially more differences across role and career stage, rather than ethnic group in relation to conference access and experience.” Another example can be found in the two abstracts pointing towards different objectives as mentioned earlier.

 We agree with the other reviewers that the letter prompts valuable reflections on organizing scientific conferences differently, which is its purpose. It would be inspiring if the authors could concretize actions and thus, make them more available to other conference organizers and/or test these in their own events. Since we also organize scientific conferences, the letter has prompted us to consider new approaches, and we share these thoughts here in the hope they will be helpful:

  

- For example, experimenting with an ambassador model where successful representatives of minoritized groups encourage, foster, and substantiate relevant networks during conferences and beyond (e.g., in pre-conference online meetings) could reduce feelings of isolation at major events.
- Another idea is to implement innovative session formats that transfer ‘tacit knowledge’ from the field, such as identifying relevant target journals and prestigious seminars, which could mitigate disadvantages for scholars not socialized in prominent labs/groups/institutes.
- Lastly, session organizers and committee members, who often serve as gatekeepers to presentations and posters, could be incentivized to ensure better balance.

To further this idea, it would be great to see how future experiments by the author team with different actions in their conferences and how these impact satisfaction and both subjective and objective barriers to accessing and benefiting from conferences.

 We hope these suggestions result useful to the authors as well as interested readers. As a final remark, we would like to suggest that it might also be beneficial to the open review process if a reviewer from a minoritized group were asked for their expertise, experience, and evaluation of this open letter.

Does the article adequately reference differing views and opinions?

Partly

Are all factual statements correct, and are statements and arguments made adequately supported by citations?

Partly

Is the Open Letter written in accessible language?

Yes

Where applicable, are recommendations and next steps explained clearly for others to follow?

Partly

Is the rationale for the Open Letter provided in sufficient detail?

Yes

Reviewer Expertise:

Susanne's research focuses on the organization of science, managing open and collaborative scientific knowledge production, Open Innovation in Science, and the role of AI in Science; Thomas has experiences in designing co-creation processes and foster public involvement in science. In addition, We have substantial experience in organizing scientific conferences.

We confirm that we have read this submission and believe that we have an appropriate level of expertise to confirm that it is of an acceptable scientific standard, however we have significant reservations, as outlined above.

# Response

**Published:** 2024-11-13

**Authors:** Ireena Dutta

**Affiliations:** Wellcome Connecting Science, Hinxton, England, UK

We thank Reviewer 3 (Susanne Beck and Thomas Palfinger) for their detailed feedback and comments, and for their description of this work as timely and relevant. Many of their comments echo those of Reviewer 2, and we have addressed the reasoning for our approach to this project and its communication (via an Open Letter). We have amended the structure of the article, to improve the presentation of the project objectives, and to link specific actions to recommendations; and hope that this will make the article a more useful resource for others. We also thank the authors for sharing their own approaches in this area, and have added additional content to build on some of these potential actions. Outside of this article, we will continue to monitor the impact (if any) of our actions and identify an appropriate route to share these. We are also open to developing a formal research project in this area. Although our intention was not to undertake original research, the feedback we have received indicates that this may be a relevant next step, and we would be happy to explore opportunities for collaboration.

# Reviewer report: Reviewer response for version 1

**DOI:** 10.21956/wellcomeopenres.23090.r83719

**Published:** 2024-07-23

**Authors:** Lyn Horn

**Affiliations:** 1 University of Cape Town, Rondebosch, Western Cape, South Africa

Exploring the experiences of minoritised groups at life sciences conferences in the UK: new perspectives and actions to improve inclusivity in the sector

 This open letter conveys the key findings and recommendations of a research report titled **Experiences of Minoritised Groups at Life Science Research Conferences in the UK** conducted by Sea-Change Consultancy on behalf of Wellcome Connecting Science conferences. The researchers conducted a study that included a desk top review, an online  survey of participants identified via the WCS data base and follow up interviews with a selection of survey participants who agreed to be interviewed.  The focus of the research was to explore and better understand the experiences of ethnic minority scholars attending conferences in the life sciences and to compare their experiences with those of their white colleagues. The study also aimed at identifying changes to practice that could improve the conference experience of ethnic minorities, if gaps were identified.  The study findings are available in a  full 60+ page report. A link to the report is provided in the open letter and hence I have also read the report to understand the context of the letter better.

 Of note, the requested review is of the open letter rather than specifically the full report. The need for equity and diversity in science was a focus at both previous (7 ^th^ and 8 ^th^) World Conferences on Research Integrity, with the conclusion drawn that a lack of both can undermine research integrity.  Much of these discussions have been centered on the roles played by  funders, publishers, research teams and institutions. An initiative to bring conferences and conference providers into the discussion is thus important and well timed. However, I do wonder why the researchers have chosen this route, a lengthy report and brief open letter, rather than a succinct research article to present their findings. I think the latter may have been more impactful.

 My task is to review the letter not the report (or that is how I have understood the request) but I would like to make a few brief comments on the report that I hope will be useful. I found the first two sections on the desktop and rapid evidence review informative and likely to be of much value to a wide range of conference organisers.

 It would have been very useful to have seen the survey data displayed more comprehensively and graphically, rather than via a narrative. This does seem to have been a relatively small sample. Was there response bias? Also, some of the numbers quoted in the survey narrative are very small and seem to reflect numbers from the interviews? “ *All respondents who attended WCS conferences and reported that Funding/bursaries from WCS had enabled them to attend were white (n=2). Whilst all respondents who self-funded their attendance at WCS conferences were from ethnically minoritised groups (n=2), despite being in the PhD/ECR stage of their career” *(4.3.2. Pg22). This may be a misinterpretation on my part but graphs and figures displaying this data would have made the information clearer.

 The Aims of the project are clearly articulated in the letter. A short description of the methodology used should be included with a brief statement on how the survey was distributed, to whom and how many, response rate, indication of response bias and recruitment procedures and selection for interviews.

 The Summary of Insights section includes a paragraph that is a bit misleading and underplays the insights gained by the study, as it contradicts itself.  It does not adequately explain the findings (which are explained in the full report), that often there is an intersection of disadvantage, or a layering that can amplify the problems experienced by ethnic minorities. This is especially so if they are also female and early career researchers.  I suggest rewording the paragraph “There were broadly no major differences between ethnically minoritised and White researchers in the discussion and responses to these questions………However, there were some notable areas of difference between groups” to better capture this nuance and lead into the differences highlighted in the bullet points that follow.

 The last section of the letter focuses on Next steps and Actions. Of note the full report contains 24 specific recommendations which have been summarised into four important bullet points. However, it would be valuable in my view, to provide a little more detail in this section, reflecting more closely the report recommendations as these will be most helpful to other conference organisers. This could be achieved by editing down the concluding paragraphs.

 In conclusion this letter is a valuable addition to current discussions around diversity and equity in all research contexts and I support its indexing.

Does the article adequately reference differing views and opinions?

Yes

Are all factual statements correct, and are statements and arguments made adequately supported by citations?

Yes

Is the Open Letter written in accessible language?

Yes

Where applicable, are recommendations and next steps explained clearly for others to follow?

Partly

Is the rationale for the Open Letter provided in sufficient detail?

Yes

Reviewer Expertise:

Health research ethics and research integrity. I was the lead author of the Cape Town statement on Fostering Research Integrity through  fairness and equity

I confirm that I have read this submission and believe that I have an appropriate level of expertise to confirm that it is of an acceptable scientific standard, however I have significant reservations, as outlined above.

# Response

**Published:** 2024-11-13

**Authors:** Ireena Dutta

**Affiliations:** Wellcome Connecting Science, Hinxton, England, UK

We thank Reviewer 2 (Lyn Horn) for their detailed feedback and comments around this project, and this article. We find it reassuring that this work is considered part a wider movement focussed on research integrity, and the role of equity and diversity within this. The reviewer asks why we have decided to share information about this work, its motivations and our response as conference developers and organisers, as a report and an Open Letter rather than a research article. We have not amended our Open Letter article in response to this comment, but would like to address it here, to provider further context for both the reviewer and other readers. We did not commission this work as a research exercise, but as a way to understand the views and perspectives of our audience(s), in order to improve our learning and training offer. By choosing a non-research project option, which is based on an understanding of our current systems and processes and the wider operating environment in the UK, we believe the outputs of this work are closer to practical application; and better positioned to deliver positive change. If we had undertaken a research project, we would have approached this differently, and delivered the results via a research publication. Having undertaken this work, primarily to benefit our own audiences, we realised that it may be useful to share our findings with the wider research community. Identifying a platform or journal that is willing to accept submissions of research-related (but not original research) content has been challenging, and we thank Wellcome Open Research for including an article format (Open Letter) that supports this type of content.

# Reviewer report: Reviewer response for version 1

**DOI:** 10.21956/wellcomeopenres.23090.r81793

**Published:** 2024-05-15

**Authors:** Sara Lil Middleton

**Affiliations:** 1 University of Oxford, Oxford, England, UK

This article seeks to examine the experiences of ethnically marginalised groups attending UK conferences with the aim to identify barriers and work towards greater conference inclusion. The article provides an overview of the report from the Wellcome Connecting Science project commissioned by Sea-Change Consultancy in 2022.

 I commend this article for bringing attention an important (and timely) aspect of academic activities. The article as a whole is well written with accessible language and the project goals and action points are generally communicated well. My comments below aim to widen the authors’ perspectives on use of language, intersectionality and provide suggestions to improve clarity of key project results.

**Abstract:**

 Reading the title, key words and abstract it is unclear that the project’s focus is on race and ethnicity (which is clearly communicated in the “Introduction and context” section). Adding a sentence in the abstract stating the project focus would help with continuity and article indexing/discoverability.

 Key result /take home message from the project findings needs to be more clearly communicated. Integrating the key findings into the last sentence “Here we discuss our….” should suffice.

**Plain Language Summary:**

 As with abstract, key results needs to be communicated more explicitly (e.g. what were the key barriers identified?)

**Introduction and context:**

 Consider using ‘racialised’ before Black and White as this intentionally challenges the perceptions of Whiteness as the neutral baseline.

 Consider using “people of the global majority” in place of “people of colour”, as this term does not essentialise people or groups and challenges the prevailing racialised White Eurocentric norms ^1^.

 “In the UK, where there is a lack of diversity amongst senior leaders”  - please clarify context of this statement. If specific to academic sector, I suggest restructuring sentence to reflect this.

 There are also compounded disadvantages for South Asian researchers in UK academia which would be important to include here and has been recognised by Wellcome’s funding scheme for Black, Bangladeshi and Pakistani researchers ^2^.

**Summary of insights section:**

 This section could be improved by adding a couple more sentences about key project methods:

 Why and how the term “ethnically minoritised” has been used here.

 How many researchers participated in the project?

 What were the main methods used (e.g. surveys, focus groups)?

 Adding a simple summary figure with key project outcomes would help readers easily access key results and make this section clearer and more of a stand-alone article to the linked full report.

**Next steps and actions:**

 “…but through its event-specific evaluation and feedback, it will actively monitor any potential change in the participant experience, or the composition of the conference delegate base…” This paragraph would benefit from briefly expanding on how monitoring outcomes are to be evaluated (e.g. what is the baseline and threshold of change?) and over what timeframe.

**Acknowledgements:**

 Given the statement “We would like to thank all the researchers who participated in this work, sharing their views and experiences…” an ethics approval statement should be mentioned somewhere as personal information was gathered for this project.

**References:**

 Overall, the article could be improved by citing a wider body of literature. As a starting point, see references 3-7 for some article suggestions that the authors can engage with which can help broaden perspectives for the “Introduction and context” section.

Does the article adequately reference differing views and opinions?

Partly

Are all factual statements correct, and are statements and arguments made adequately supported by citations?

Yes

Is the Open Letter written in accessible language?

Yes

Where applicable, are recommendations and next steps explained clearly for others to follow?

Partly

Is the rationale for the Open Letter provided in sufficient detail?

Yes

Reviewer Expertise:

Justice, equity, diversity and inclusion in STEM and academia; intersectionality, bias, discrimination in academia with focus on race, gender, disability, and neurodiversity.

I confirm that I have read this submission and believe that I have an appropriate level of expertise to confirm that it is of an acceptable scientific standard, however I have significant reservations, as outlined above.

## References

[1] : Outcomes Following Pedestal Cup Reconstruction of (Impending) Pathological Fractures of the Acetabulum due to Metastatic Bone Disease. *Indian J Surg Oncol* .2024;15(2) : 10.1007/s13193-024-01917-x 428-436 10.1007/s13193-024-01917-x 38741639 PMC11088586

[2] Reference source

[3] Reference source

[4] : “Which door should I go through?” (In)visible intersections of race and disability in the academy. *Area* .2020;52(4) : 10.1111/area.12554 687-694 10.1111/area.12554

[5] : Carefree conferences? Academics with caring responsibilities performing mobile academic subjectivities. *Gender and Education* .2020;32(1) : 10.1080/09540253.2019.1685654 70-85 10.1080/09540253.2019.1685654

[6] : Ten simple rules to host an inclusive conference. *PLOS Computational Biology* .2022;18(7) : 10.1371/journal.pcbi.1010164 10.1371/journal.pcbi.1010164 PMC930273235862309

[7] : (dis-)Belonging bodies: negotiating outsider-ness at academic conferences. *Gender, Place & Culture* .2020;27(6) : 10.1080/0966369X.2019.1609913 765-787 10.1080/0966369X.2019.1609913

# Response

**Published:** 2024-11-13

**Authors:** Ireena Dutta

**Affiliations:** Wellcome Connecting Science, Hinxton, England, UK

We thank Reviewer 1 (Sara Lil Middleton) for their feedback and positive and constructive comments, particularly around the use of language and additional references, which have fed into our own knowledge base. We agree that language is a powerful tool in relation to inclusion, and should be carefully considered. We have therefore included a new section to reflect the context of our decision-making around the use of language to describe characteristics and identities. We have also amended the structure of the article to improve its clarity, which addresses similar feedback from Reviewer 3.
